# A Study on the Effect of Piezoelectric Nonlinearity on the Bending Behaviour of Smart Laminated Composite Beam

**DOI:** 10.3390/ma16072839

**Published:** 2023-04-02

**Authors:** Adnan Akhlaq, Mohd Sultan Ibrahim Shaik Dawood, Mohamed Ali Jaffar Syed, Erwin Sulaeman

**Affiliations:** 1 Department of Mechanical and Aerospace Engineering, Kulliyyah of Engineering, International Islamic University Malaysia, Kuala Lumpur 53100, Malaysiajaffar@iium.edu.my (M.A.J.S.); 2 Department of Mechanical and Aerospace Engineering, College of Engineering, United Arab Emirates University, Al-Ain P.O. Box 15551, United Arab Emirates; esulaeman@uaeu.ac.ae

**Keywords:** elastostriction, electrostriction, piezoelectric, composite beam

## Abstract

This paper presents a finite element analysis to model and analyze composite laminated beams with distributed piezoelectric actuators attached to the top and bottom surfaces considering nonlinear constitutive equations under a high electric field. The static response is presented for piezoelectric composite laminated beam using higher order electric field nonlinearity to assess the effect of electrostriction and elastostriction coefficient at a high electric field. A finite element approach based on higher-order shear deformation theory is applied for static analysis of composite laminated beams, varying the thickness and orientation of laminates, to verify the nonlinear effect under a high electric field. A good comparison of results is shown with the available results in the literature. The finding of the results highlights the importance of considering elastostriction term along with the electrostriction term in determining the deflection and stresses of the composite laminated beam.

## 1. Introduction

Smart materials have attracted researchers due to their wide range of applications in engineering fields such as shape, vibration, noise and position control, health monitoring, and damage detection [1,2]. Piezoelectric materials have electrical and mechanical properties and they convert electrical energy into mechanical energy, i.e., it produces mechanical strain on electrical voltage (reverse piezoelectric effect) and conversely converts mechanical energy into electrical energy, i.e., develop charge upon stressed (direct piezoelectric effect). Piezoelectric materials can be used in smart structures as sensors and actuators based on direct and converse piezoelectric effects, respectively [3,4,5].

Piezoelectric materials show weak electromechanical coupling at a low electric field and hence they can be modelled using linear constitutive equations and assuming strain to be linearly dependent on the electric field. However, on applying a large electric field, the electro-elastic properties of piezoelectric materials behave nonlinearly. The nonlinear strain equation was derived by Joshi [6], including the electrostriction and the elastostriction. Tiersten [7] presented rotationally invariant nonlinear electro-elastic equations for applying large electric fields with significant strains. Wang et al. [8] studied the nonlinear electromechanical behaviour of cantilevered piezoelectric ceramic unimorph and bimorph beams. Yao et al. [9] studied the nonlinear characteristics of cantilever piezoelectric actuators using Classical Laminate Theory. Thornburgh & Chattopadhyay [10] used Higher-order Shear Deformation Theory to obtain the nonlinear behaviour of the piezoelectric actuator.

Wischke et al. [5] experimented with nonlinear electrostrictive and elastostrictive coefficients using a PZT cantilever unimorph. In contrast, Kapuria & Yasin [11] presented a layerwise finite element model for the static analysis of piezoelectric laminates considering nonlinear electrostrictive coefficient under strong electric field and extended the model to vibration control of plate and shell [12,13]. Analysis of piezoelectric cantilever bimorph is conducted by Chattaraj & Ganguli [14] using electro-elastic nonlinearity under a strong electric field based on Euler beam theory. Recently, Sumit et al. [15] developed an analytical model to study the effect of nonlinear coefficients on the response of unimorph and bimorph actuators using a second-order constitutive equation. Chattaraj & Ganguli [16] studied the performance improvement of piezoelectric cantilever bimorph by altering its geometry using electro-elastic nonlinearity under a strong electric field. Experimental analysis for assessing the dynamic performance of piezoelectric cantilever bimorph considering material nonlinearity is presented by Ahoor et al. [17]. Zhang et al. [18] presented an accurate modelling of a piezoelectric laminated composite structure using geometrically nonlinearity and the electroelastic material nonlinear effect at a high electric field.

Koutsawa et al. [19] presented a one-dimensional finite element model for static analysis of a composite beam with a piezoelectric layer considering non-classical effects accounted by higher-order displacement-based theories. Koutsawa et al. [20] extended the finite element model for presenting a free vibration analysis of piezoelectric layers or patches on composite beams.

The present work aims at studying the static analysis of piezoelectric composite laminated beams under high electric fields using higher-order shear deformation theory. An efficient nonlinear model is used for analysis, considering nonlinear electrostrictive and elastostrictive coefficients. Finite element formulation was used to consider a linearly varying electric potential through the thickness. The static analysis of piezo-actuated composite laminated beam is carried out and the results are validated with the available experimental and numerical results.

## 2. Mathematical Formulation

This section presents the mathematical formulation of the piezoelectric constitutive equation.

### 2.1. Theoretical Formulation Using HSDT

**Figure 1:**
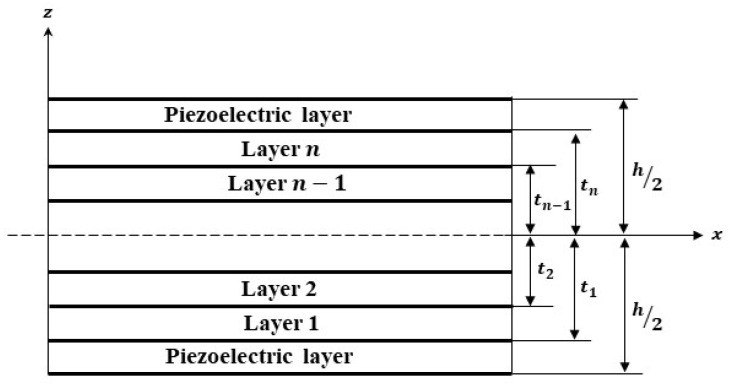
Composite laminate with a bonded piezoelectric layer at the top and bottom.

Considering the higher order shear deformation model given by Reddy [21], the displacement field equations for the composite laminated beam at any point through the thickness are presented by:

(1) $$u\left(x,y,z\right)=z\varphi_{x}-\frac{4z^{3}}{3h^{2}}(\varphi_{x}+\frac{\partial w}{\partial x})\;w\left(x,y,z\right)=w_{0}(x)$$

where $w_{0}$ denotes the displacements at any point (x,y,0) on mid-surface and $\varphi_{x}$ is the rotation angle along the x-axis. For a one-dimensional cantilever bimorph beam, as shown in Figure 1, where the width in the y-direction is small as compared to length, by using the plane stress assumption, the strain components $\varepsilon_{xx}\;and\;\gamma_{xz}$ are represented by:

(2) $$\varepsilon_{xx}=\frac{\partial u}{\partial x}=z\frac{\partial \varphi_{x}}{\partial x}-\frac{4z^{3}}{3h^{2}}(\frac{\partial \varphi_{x}}{\partial x}+\frac{\partial^{2}w}{\partial^{2}x})\;\gamma_{xz}=\frac{\partial w}{\partial x}+\varphi_{x}-\frac{4z^{2}}{h^{2}}(\varphi_{x}+\frac{\partial w}{\partial x})$$

### 2.2. Nonlinear Constitutive Relation

The nonlinear strain is given by Joshi [6] using thermodynamic Gibbs potential and Taylors series expansion up to the second order:

(3) $$\varepsilon_{ij}=S_{ijlm}^{E}\sigma_{lm}+d_{ijn}E_{n}+\frac{1}{2}S_{ijlmpq}^{E}\sigma_{lm}\sigma_{pq}+\frac{1}{2}d_{ijnr}E_{n}E_{r}+\kappa_{ijlmn}\sigma_{lm}E_{n}\;D_{k}=d_{klm}\sigma_{lm}+\epsilon_{knr}^{\sigma }E_{n}+\frac{1}{2}\kappa_{klmpq}\sigma_{lm}\sigma_{pq}+\frac{1}{2}\epsilon_{knr}^{\sigma }E_{n}E_{r}+d_{klmn}\sigma_{lm}E_{n}$$

where $\varepsilon_{ij}$ is the induced strain, $\sigma_{lm}$ is the stress in the piezoelectric component, $E_{n}$ is the applied electric field, $S_{ijlm}^{E}$ is the elastic compliance, $d_{ijn}$ piezoelectric strain constant, $S_{ijlmpq}^{E}$ is the nonlinear elastic compliance, $d_{ijnr}$ is the electrostriction coefficient, $\kappa_{ijlmn}$ is the elastostriction coefficient and $\epsilon_{knr}^{\sigma }$ is a nonlinear dielectric permittivity coefficient. 

Using the nonlinear strain expression, the plane strain equation is given as:

(4) $$[\begin{array}{c} \varepsilon_{xx}\\ \gamma_{xz} \end{array}]= [\begin{array}{cc} S_{xx} & 0\\ 0 & S_{xz} \end{array}] [\begin{array}{c} \sigma_{xx}\\ \tau_{xz} \end{array}]+ [\begin{array}{c} d_{zx}\\ 0 \end{array}]E_{z}+\frac{1}{2} [\begin{array}{c} d_{zzx}\\ 0 \end{array}]E_{z}^{2}+ [\begin{array}{c} \kappa_{zzx}\\ 0 \end{array}] [\begin{array}{c} \sigma_{xx}\\ \tau_{xz} \end{array}]E_{z}\;D_{z}= [\begin{array}{cc} 0 & 0\\ d_{zx} & 0 \end{array}] [\begin{array}{c} \sigma_{xx}\\ \tau_{xz} \end{array}]+\epsilon_{zz}E_{z}+\frac{1}{2}\epsilon_{zz}E_{z}^{2}+ [\begin{array}{cc} 0 & 0\\ d_{zzx} & 0 \end{array}] [\begin{array}{c} \sigma_{xx}\\ \tau_{xz} \end{array}]E_{z}$$

(5) $${ [\begin{array}{c} \sigma_{xx}\\ \tau_{xz}\\ D_{z} \end{array}]}_{k}={ [\begin{array}{c} Q_{11}\\ 0\\ e_{zx} \end{array}\begin{array}{c} 0\\ Q_{55}\\ 0 \end{array}\begin{array}{c} 0\\ 0\\ 0 \end{array}]}_{k}{ [\begin{array}{c} \varepsilon_{xx}\\ \gamma_{xz}\\ 0 \end{array}]}_{k}-{ [\begin{array}{c} e_{zx}\\ 0\\ \epsilon_{zz} \end{array}]}_{k}E_{z}$$

where:

$$S_{xx}=\frac{1}{Y_{xx}}\;\;\;\;\;\;and\;\;\;\;S_{xz}=\frac{1}{G_{xz}}\;Q_{11}=\frac{1}{S_{xx}+\kappa_{zzx}E_{z}},Q_{55}=G_{xz}\;\;\;\;\;\;and\;\;\;\;e_{zx}=\frac{d_{zx}+\frac{1}{2}d_{zzx}E_{z}}{S_{xx}+\kappa_{zzx}E_{z}}$$

$Y_{xx}$ and $G_{xz}$ are the elastic constants, $S_{xx}\;and\;S_{xz}$ are the elastic compliances. $Q_{11}$ and $e_{zx}$ are the nonlinear material stiffness constant and nonlinear piezoelectric constant of the piezoelectric actuator dependent on the applied electric field respectively while $\kappa_{zzx}$ is the elastostriction coefficient and $d_{zzx}$ is the electrostriction coefficient. 

### 2.3. Energy Formulation

The total internal strain energy for the piezoelectric structure U is expressed as the sum of mechanical strain energy and the electric field potential energy as [22]

(6) $$U=\frac{1}{2}\int_{v}\sigma \epsilon d\Omega -\frac{1}{2}\int_{v}E_{3}Ddv$$

where *v* is the volume of the element. Substituting Equation (5) into Equation (6), the total strain energy of a piezoelectric structure is written as:

(7) $$U=\frac{1}{2}\int_{V}[\left(\sigma_{xx}\varepsilon_{xx}+\tau_{xz}\gamma_{xz}\right)-\left(D_{z}E_{z}\right)]dv\;U=\frac{1}{2}\int_{V} [(Q_{11}\varepsilon_{xx}-e_{zx}E_{z})\varepsilon_{xx}+{(Q}_{55}\gamma_{xz}{)\gamma }_{xz}-{(e}_{zx}\varepsilon_{xx}+\epsilon_{zz}E_{z}{)E}_{z}]dv\;U=\frac{1}{2}\int_{v}(Q_{11}{\varepsilon_{xx}}^{2}+Q_{55}{\gamma_{xz}}^{2}-2e_{zx}E_{z}\varepsilon_{xx}-\epsilon_{zz}{E_{z}}^{2})dv$$

### 2.4. Finite Element Formulation

A two-node finite element with three mechanical degrees of freedom $\left(w,\frac{dw}{dx},\varphi_{x}\right)$ is considered for analysis. A linear shape function is used to represent the rotation angle at the mid-surface ${(\varphi }_{x})$:

(8) $$\varphi_{x}(x)=a_{0}+a_{1}x$$

Solving for end conditions, the rotation angle at the mid-surface ${(\varphi }_{x})$ is represented in terms of nodal displacement as:

(9) $$\varphi_{x}=N_{1}{\varphi_{x}}_{1}+N_{2}{\varphi_{x}}_{2}$$

where:

$$N_{1}=1-\frac{x}{l}\;\;\;\;\;and\;\;\;\;\;\;\;\;\;\;N_{2}=\frac{x}{l}$$

A Hermite cubic shape function is used to represent the transverse deflection (w):

(10) $$w\left(x\right)=a_{0}+a_{1}x+a_{2}x^{2}+a_{3}x^{3}\;\theta =\frac{\partial w}{\partial x}=a_{1}+2a_{2}x+3a_{3}x^{2}$$

Solving Equation (10) using the end conditions, the transverse deflection (w) is represented in terms of nodal displacement as:

(11) $$w=N_{3}w_{1}+N_{4}\theta_{1}+N_{5}w_{2}+N_{6}\theta_{2}$$

where:

$$N_{3}=1-3\frac{x^{2}}{l^{2}}+2\frac{x^{3}}{l^{3}},\;\;\;\;\;N_{4}=x-2\frac{x^{2}}{l}+\frac{x^{3}}{l^{2}}\;N_{5}=3\frac{x^{2}}{l^{2}}-2\frac{x^{3}}{l^{3}},\;N_{6}=-\frac{x^{2}}{l}+\frac{x^{3}}{l^{2}}$$

The axial strain due to bending is written in terms of shape function as:

(12) $$\varepsilon_{xx}= [\begin{array}{cc} z & -\frac{4z^{3}}{3h^{2}} \end{array}] [\begin{array}{c} \begin{array}{c} \frac{\partial \varphi_{x}}{\partial x}\\ \frac{\partial \varphi_{x}}{\partial x}+\frac{\partial^{2}w}{\partial^{2}x} \end{array} \end{array}]$$

The shear strain due to shear is written in terms of shape function as:

(13) $$\gamma_{xz}=\frac{\partial w}{\partial x}+\varphi_{x}-\frac{4z^{2}}{h^{2}}\left(\varphi_{x}+\frac{\partial w}{\partial x}\right)\;\gamma_{xz}= [\begin{array}{cc} 1 & -\frac{4z^{2}}{h^{2}} \end{array}] [\begin{array}{cccccc} \frac{\partial N_{3}}{\partial x} & \frac{\partial N_{4}}{\partial x} & N_{1} & \frac{\partial N_{5}}{\partial x} & \frac{\partial N_{6}}{\partial x} & N_{2}\\ \frac{\partial N_{3}}{\partial x} & \frac{\partial N_{4}}{\partial x} & N_{1} & \frac{\partial N_{5}}{\partial x} & \frac{\partial N_{6}}{\partial x} & N_{2} \end{array}]\left\{\begin{array}{c} \begin{array}{c} w_{1}\\ \theta_{1} \end{array}\\ {\varphi_{x}}_{1}\\ \begin{array}{c} \begin{array}{c} w_{2}\\ \theta_{2} \end{array}\\ {\varphi_{x}}_{2} \end{array} \end{array}\right\}\;\gamma_{xz}= [\begin{array}{cc} 1 & -\frac{4z^{2}}{h^{2}} \end{array}] [C]d_{e}$$

The electric field across the thickness of the piezoelectric layer is given as a linear function of the electric potential ∅ and can be expressed in matrix form as:

(14) $$[E_{z}]= [-\frac{\emptyset }{t}]\; [E_{z}]= [B_{\emptyset }][\emptyset ]$$

where:

$$[B_{\emptyset }]= [\frac{-1}{t}]$$

### 2.5. Principle of Virtual Work

The principle of minimum potential energy that follows the virtual work principle is used to obtain the stiffness matrices. The total potential energy for the piezoelectric element is given as:

(15) $$\prod =\sum U-\sum fd_{e}-\int_{A}\phi QdA$$

For minimum potential the total potential energy does not vary, having variation concerning nodal displacement as zero:

(16) $$\frac{\partial \prod }{\partial d_{e}}=\frac{\partial }{\partial d_{e}}\sum U-\frac{\partial }{\partial d_{e}}\sum fd_{e}=0$$

Solving this equation, we obtain the stiffness matrices for bending, shear, and electrical load as:

(17) $${ [K^{e}]}_{b}=Q_{11}\int_{v}{ [B]}^{T}{ [\begin{array}{cc} z & -\frac{4z^{3}}{3h^{2}} \end{array}]}^{T} [\begin{array}{cc} z & -\frac{4z^{3}}{3h^{2}} \end{array}] [B]dv\;{ [K^{e}]}_{b}=Q_{11}\int_{0}^{l}{ [B]}^{T}I [B]dx$$

(18) $${ [K^{e}]}_{s}=Q_{55}\int_{v}{ [C]}^{T}{ [\begin{array}{cc} 1 & -\frac{4z^{2}}{h^{2}} \end{array}]}^{T} [\begin{array}{cc} 1 & -\frac{4z^{2}}{h^{2}} \end{array}] [C]dv\;{ [K^{e}]}_{s}=Q_{55}\int_{0}^{l}{ [C]}^{T}I^{\prime } [C]dx$$

(19) $${ [K^{e}]}_{\emptyset }={-e}_{31}\int_{v}{ [B]}^{T}{ [\begin{array}{cc} z & -\frac{4z^{3}}{3h^{2}} \end{array}]}^{T} [B_{\emptyset }]dv\;{ [K^{e}]}_{\emptyset }={-e}_{31}\int_{v}{ [B]}^{T} [\begin{array}{c} I_{1}\\ -\frac{4I_{3}}{3h^{2}} \end{array}] [B_{\emptyset }]dx$$

where

$$I= [\begin{array}{cc} I_{2} & -\frac{4I_{4}}{3h^{2}}\\ -\frac{4I_{4}}{3h^{2}} & \frac{16I_{6}}{9h^{4}} \end{array}],\;\;\;\;\;I^{\prime }= [\begin{array}{cc} I_{0} & -\frac{4I_{2}}{h^{2}}\\ -\frac{4I_{2}}{h^{2}} & \frac{16I_{4}}{h^{4}} \end{array}]\;{(I}_{0},I_{1},I_{2},I_{3},I_{4},I_{5},I_{6})=b\int_{h_{k}}^{h_{k+1}}\left(1,z,z^{2},z^{3},z^{4},z^{5},z^{6}\right)dz$$

Considering three mechanical degrees of freedom and one electrical degree of freedom the elemental stiffness matrices will be meshed to form a global stiffness matrix. The equation of motion in the case of piezoelectric actuation only will be given in terms of the global stiffness matrix as:

(20) $${\{ [K]}_{b}+{ [K]}_{s}\} [w]={ [K]}_{\emptyset }[\emptyset ]$$

where ${ [K]}_{b},{ [K]}_{s}\;and\;{ [K]}_{\emptyset }$ are global stiffness matrices for bending, shear, and electrical load, respectively. Solving Equation (20), we can obtain the deflection at each node which will be used to perform the stress analysis.

## 3. Results and Discussion

### 3.1. Validation of Results with Unimorph and Bimorph with Linear Piezoelectric Coefficients

**Figure 2:**
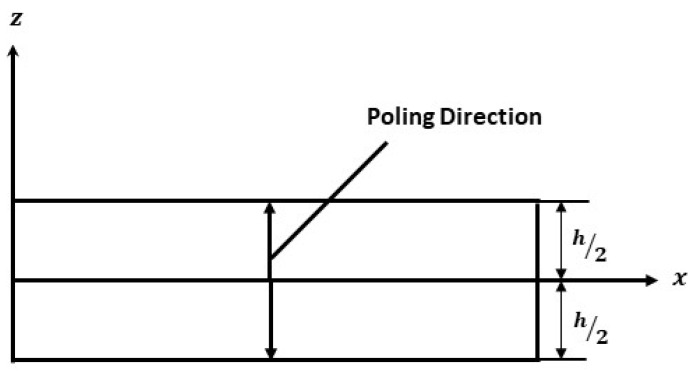
Cantilever Bimorph Beam with opposite polarity.

To validate the present model a PVDF bimorph beam considered by researchers Jiang & Li [4], Hwang & Park [23] and Tzou & Ye [24] is taken, as shown in Figure 2. A two-layer bonded cantilever bimorph is considered with opposite polarities. The dimensions of the beam are 100 mm×5 mm×1 mm. When a unit voltage is applied across the thickness of the beam i.e., 0.5 V at the top layer and −0.5 V at the bottom layer, the induced strain will produce bending of the beam. Five beam elements of equal length are used to calculate the transverse deflections at each node. The properties of the PVDF actuator are given in Table 1. The transverse deflection results found using current FEM and results of Jiang & Li [4] using the theoretical equation of the principle of minimum potential energy are presented in Table 2 and compared with the previously published results. 

The present model results with linear piezoelectric properties agree well with Tzou & Ye [24], which used finite elements with First-order Shear Deformation Theory (FSDT), whereas Jiang & Li [4] used Third-order Shear Deformation Theory. 

**Figure 3:**
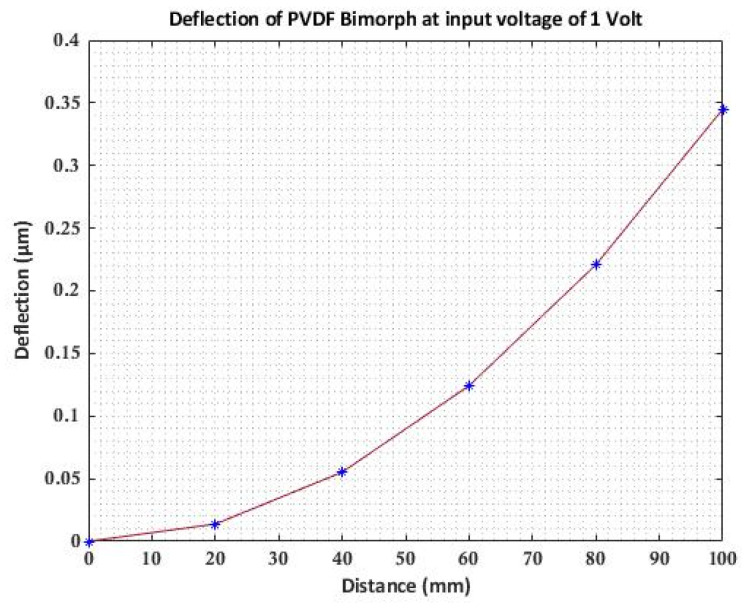
Deflection of PVDF bimorph cantilever beam.

**Figure 4:**
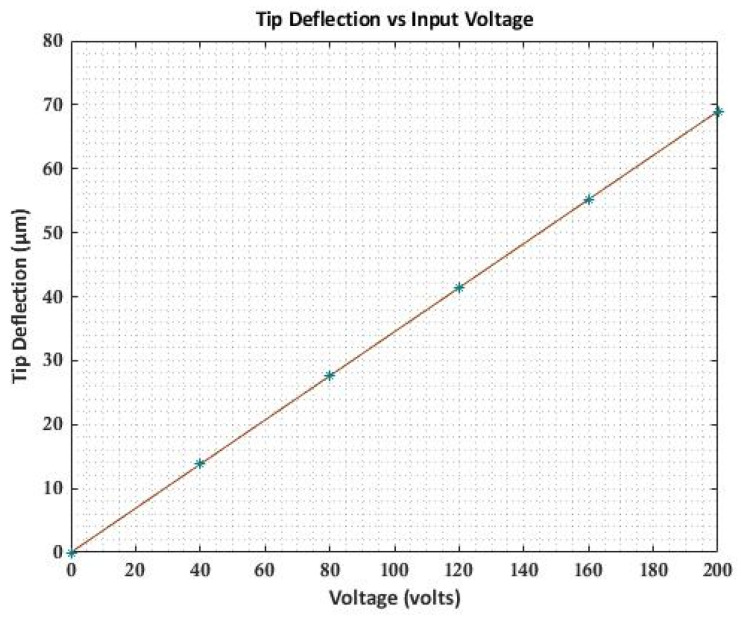
Variation of deflection with input voltage.

The deflection of PVDF bimorph at an input voltage of 1 Volt is shown in Figure 3, and the variation of transverse deflection with input voltage varying from (0–200 V) is shown in Figure 4. The transverse deflection shows linear variation with a varying input voltage as the linear constitutive equation is used. 

**Figure 5:**
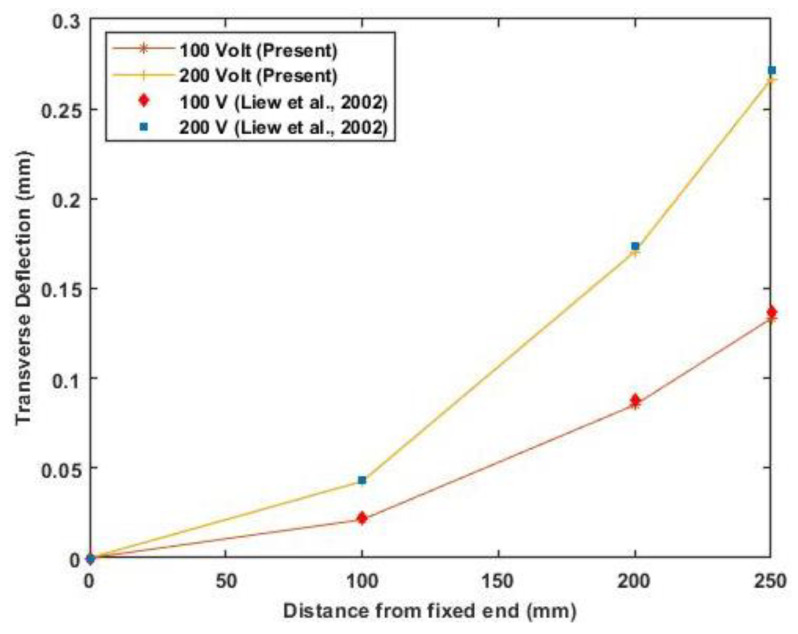
Transverse deflection of the cantilever beam for 100 and 200 volts (Reference results are taken from Liew et al. [25]).

**Figure 6:**
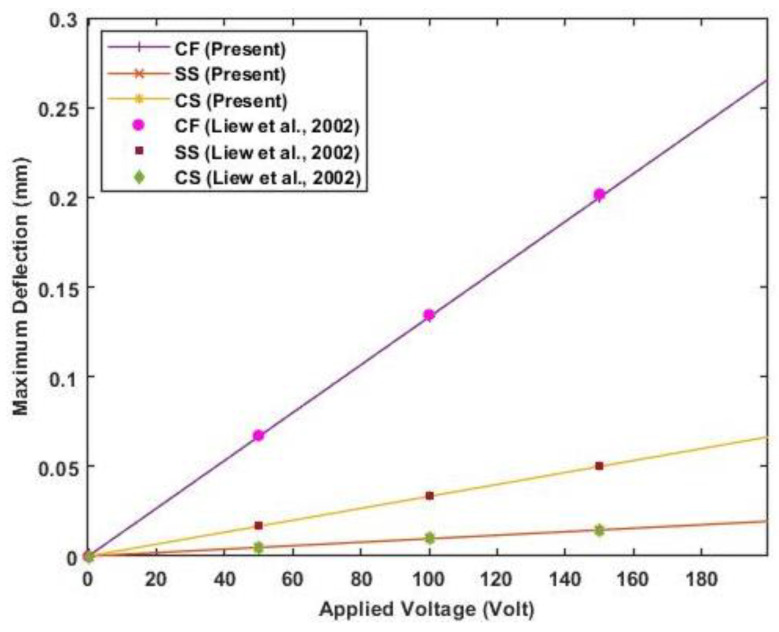
The maximum deflection of the beam for different end conditions against actuator voltage (Reference results are taken from Liew et al. [25]).

For the analysis of the composite laminated beam, consider a graphite-epoxy laminated composite beam with piezoelectric actuation, with the material properties as listed in Table 3. A layer of piezoceramic material is surface bonded to the top and bottom of the symmetric cross-ply laminated beam of orientation ($0^{{}^{\circ}}/90^{{}^{\circ}}/90^{{}^{\circ}}/0^{{}^{\circ}}$). The top layer is polarized in the same direction as the applied voltage, while the bottom layer is polarized in the opposite direction of the applied voltage. The dimension of each ply is 0.254 m×0.0254 m×0.00127 m, and the thickness of the PZT actuator is 0.0002 m. Figure 5 shows the deflection of the cantilever beam when actuated at 0, 100, and 200 volts. In contrast, Figure 6 presents the maximum deflection against applied voltage for different end conditions (clamped-free, clamped-simply supported, and simply supported).

The results of transverse deflection for the cantilever beam and maximum deflection of the beam with different end conditions agree well with previously published results. Figure 6 shows a linear variation of maximum deflection with applied voltage as a linear constitutive equation is used. 

**Figure 7:**
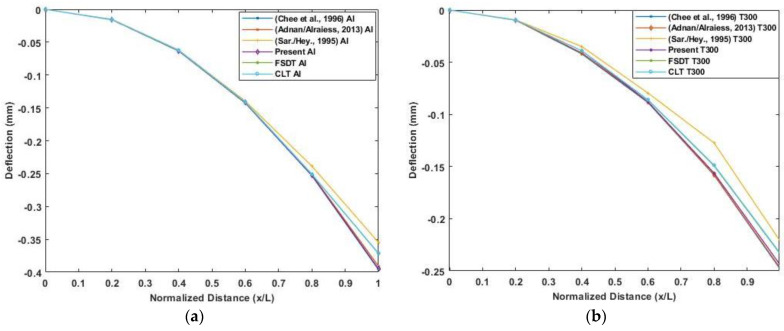
Deflection by piezoelectric actuation along the normalized length of the cantilever beam: (**a**) Aluminum beam; (**b**) Gr/epoxy composite beam. (Reference results are taken from Adnan Alraiess [22], Chee et al. [26] and Saravanos and Heyliger’s [27]).

A three-layered cantilever beam made up of elastic substrate, adhesive, and piezoelectric material is used to verify the model. The dimension of the beam is 152.4 mm×2.54 mm×17 mm. A 12.5 kV actuator voltage is applied across the thickness of piezoelectric material. The elastic substrate is made of isotropic aluminum or Gr/Epoxy composite T300/934 (0°). The relevant material properties are given in Table 3. The structure is divided into five equal-length beam elements. Figure 7 presents the results of the transverse deflection of beam for aluminum and Gr/Epoxy using the present model compared to Classical laminate theory, First order shear deformation theory, and the existing literature results. The transverse deflection results using the present model show good agreement of results with Adnan Alraiess [22] and Chee et al. [26] and as both using Higher order shear deformation theory as compared to Saravanos and Heyliger’s [27] layer-wise model. 

### 3.2. Validation of Nonlinear Analysis of Piezoelectric Cantilever Bimorph and Unimorph

A PZT cantilever bimorph of 35×7×0.5 mm dimension with opposite polarity is considered as shown in Figure 2 to validate the effect of the electrostrictive coefficient on the deflection of a beam. The example is numerically analyzed by researchers [11,14] using the effect of the electrostrictive coefficient in the nonlinear constitutive equation and validated the results for transverse deflection with the experimental results of Wang et al. [8]. 

**Figure 8:**
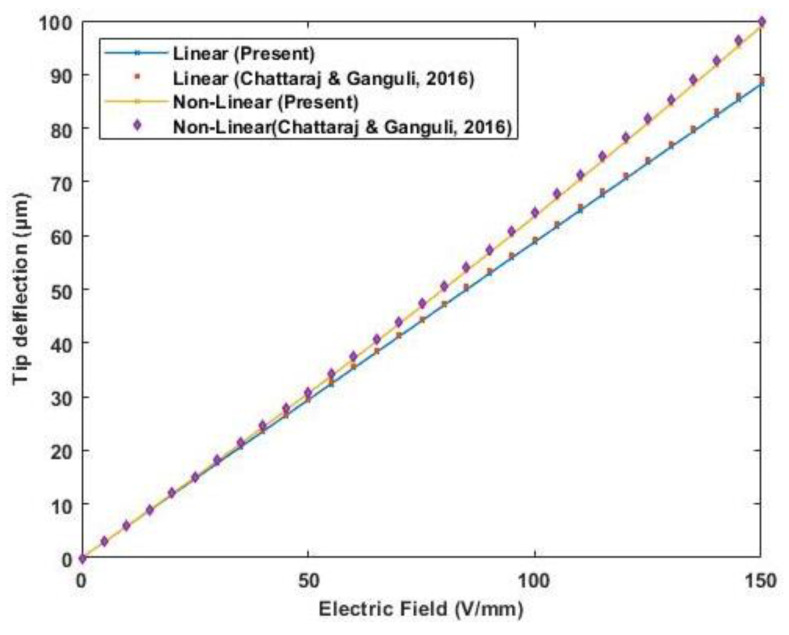
Variation of tip deflection with the increasing electric field for cantilever bimorph with opposite polarity (Reference results are taken from Chattaraj & Ganguli [14]).

The properties of the PVDF actuator are provided in Table 4. A plot of transverse deflection is presented in Figure 8, considering linear and nonlinear constitutive equations based on the developed model. The results of transverse deflection using the present model show good accuracy of results with previously published results. 

**Figure 9:**
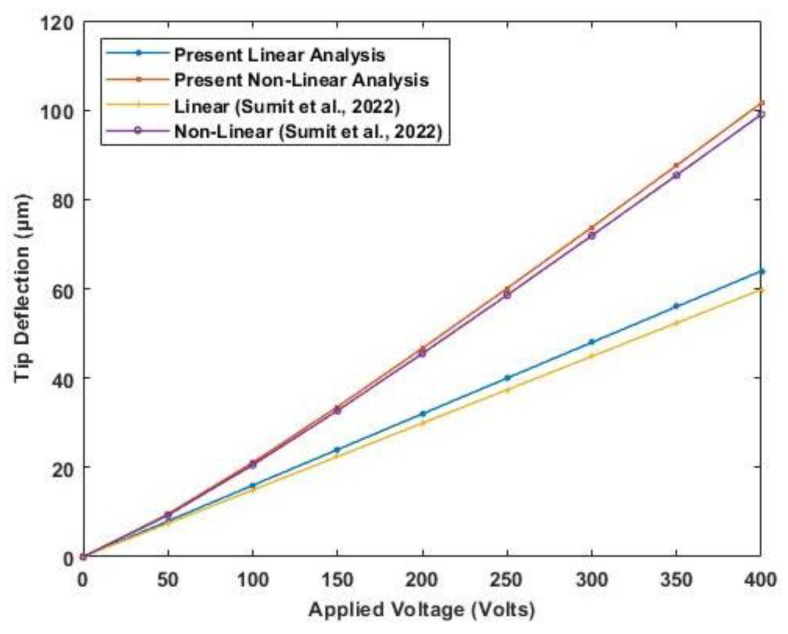
Variation of tip deflection with the increasing electric potential for cantilever unimorph (Reference results are taken from Sumit et al. [15].

A PZT (APC 850) cantilever unimorph of 23×3 mm is used to validate the nonlinear effect of both electrostriction and elastostriction coefficient on the deflection of the beam. A piezoelectric unimorph with a 0.3 mm thick piezoelectric actuator and 0.675 mm thick silicon as an elastic layer. The properties of the PZT actuator and silicon are provided in Table 4. The tip deflection based on linear piezoelectric relation and nonlinear coefficients is plotted in Figure 9. The result obtained compared well with the results presented by Sumit et al. [15]. 

### 3.3. Nonlinear Analysis of Piezo-Actuated Laminated Composite Beam

Using symmetric cross-ply and anti-symmetric angle-ply laminated composite beams with different end conditions, the effect of nonlinear parameters, i.e., electrostriction coefficient and elastostriction coefficient, is studied.

**Figure 10:**
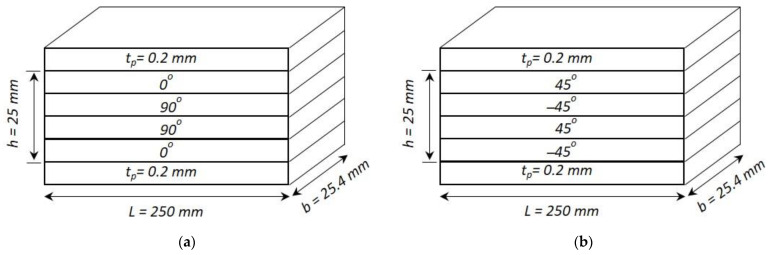
Composite laminated beam: (**a**) symmetric cross-ply laminate; (**b**) anti-symmetric angle-ply laminate configuration (*a*/*h* = 10).

**Figure 11:**
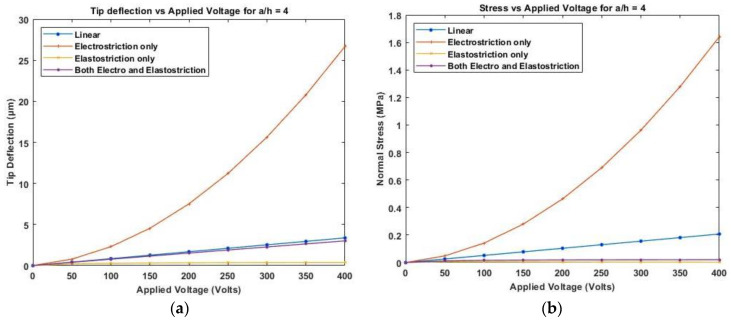
Variation of (**a**) deflection and (**b**) normal stress for a thick beam with the applied voltage for symmetric cross-ply laminate.

A symmetric cross-ply composite beam with four layers of a graphite-epoxy substrate (0°/90°/90°/0°) and an anti-symmetric angle-ply composite beam with four layers of a graphite-epoxy substrate (45°/−45°/45°/−45°) each elastic layer of 0.25 h thick, with two PZT outer layers each of 0.2 mm thick, as shown in Figure 10. In both cases, a 250 mm long beam with a thickness ratio of a/h=4,10, and 100 is evaluated and modeled with three mechanical degrees of freedom and one electrical degree of freedom at each node. The material properties are given in Table 4, whereas Sumit et al. [15] provided the nonlinear parameters as $d_{331}=-1210\times 10^{-18}\;m^{2}V^{-2}\;\mathrm{and}\;\kappa_{331}=-6.3\times 10^{-17}\;m^{3}N^{-1}V^{-1}.$ Figure 11 presents the variation of linear and nonlinear deflection and in-plane normal stresses for a cross-ply cantilever thick beam, a/h=4, when an electric potential is increased from 0 to 400 V is applied across the beam.

**Figure 12:**
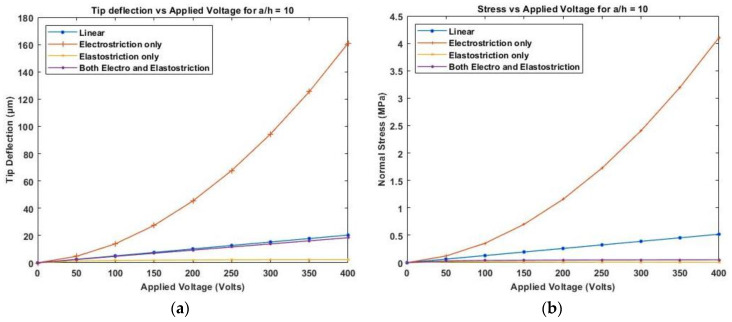
Variation of (**a**) deflection and (**b**) normal stress for a moderately thick beam with the applied voltage for symmetric cross-ply laminate.

**Figure 13:**
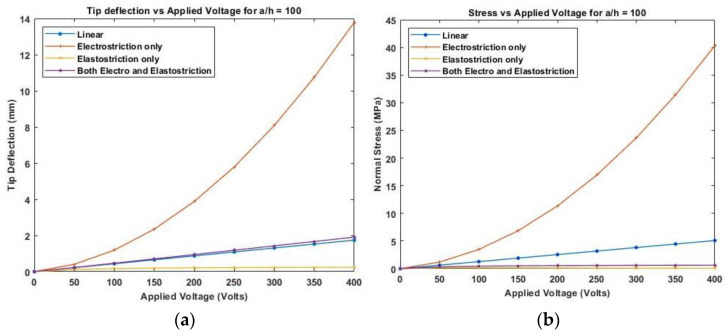
Variation of (**a**) deflection and (**b**) normal stress for a thin beam with the electric potential for symmetric cross-ply laminate.

Figure 12 presents the variation of linear and nonlinear deflection and in-plane normal stresses for a cross-ply cantilever thick beam, a/h=10 when the electric potential is increased from 0 to 400 V, whereas Figure 13, shows a similar variation of linear and nonlinear deflection and in-plane normal stresses for a cross-ply cantilever thick beam, a/h=100.

**Figure 14:**
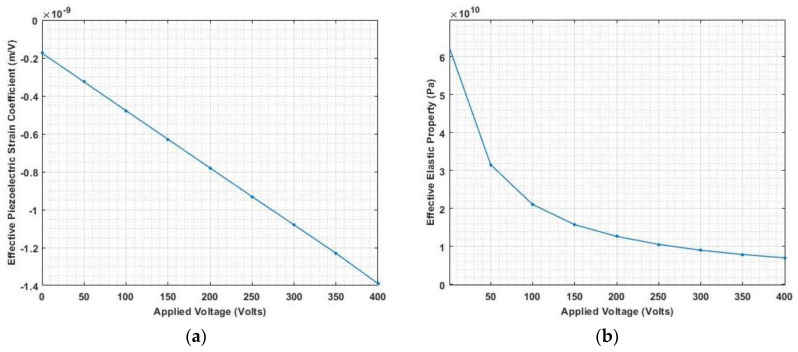
Variation of (**a**) effective piezoelectric strain coefficient and (**b**) effective elastic property with applied electric potential.

Figure 11, Figure 12 and Figure 13 show the nonlinear deflection and normal stress at the upper surface of the piezoelectric structure considering electrostriction alone, elastostriction alone, and both the nonlinear terms and it shows that taking into account both elastostriction and the electrostriction coefficient yields more accurate results. Furthermore, including the elastostriction term, the elastic characteristics of the piezoelectric material are reduced in a positive electric field, causing a decrease in deflection and stresses. In the case of a negative electric field, the deflection and stresses created by elastostriction will increase elastic property, resulting in higher deflection and stresses. The results show that using a higher-order nonlinear constitutive equation, and the effective elastic property given by Equation (5), which may increase or decrease with the positive or negative electric field and effective piezoelectric strain coefficient as $d_{zx}^{e}=d_{zx}+\frac{1}{2}d_{zzx}\left|E_{z}\right|$ increase with positive or negative electric field gives the correct response as studied by Kapuria & Yasin [11]. The absolute value of the electric field is considered because the total strain produced in the structure is quadratically dependent on the electric field with the electrostriction coefficient. The variation of effective elastic property and effective piezoelectric strain coefficient with increasing electric potential is shown in Figure 14. 

**Figure 15:**
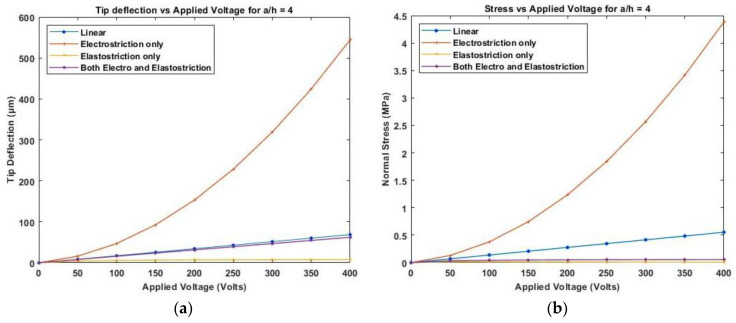
Variation of (**a**) deflection and (**b**) normal stress with electric field for thick anti-symmetric angle-ply laminate.

Figure 15 shows the variation of linear and nonlinear deflection and in-plane normal stresses for piezoelectrically actuated thick anti-symmetric angle-ply laminated beam when a positive electric potential is applied across the piezoelectric layer. The results suggest that incorporating the elastostriction coefficient improves deflection and stress accuracy. Compared to the electrostriction coefficient alone, the elastostriction term affects the flexural rigidity whereby a positive electric potential result in decreased flexural rigidity while a negative electric potential increases the flexural rigidity. As the bending stiffness of symmetric laminates is greater than the anti-symmetric laminate, deflection for anti-symmetric angle-ply laminate is greater than symmetric cross-ply laminate. 

**Figure 16:**
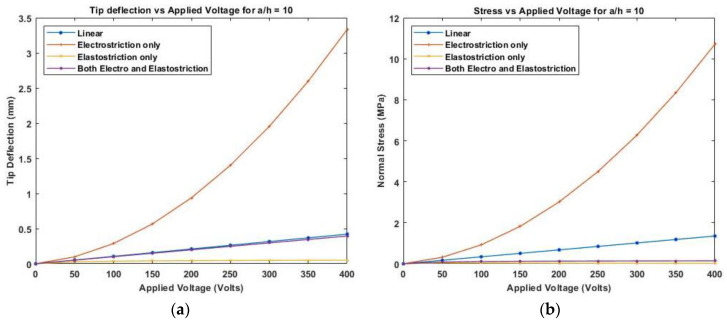
Variation of (**a**) deflection and (**b**) normal stress with electric field for moderately thick anti-symmetric angle-ply laminate.

**Figure 17:**
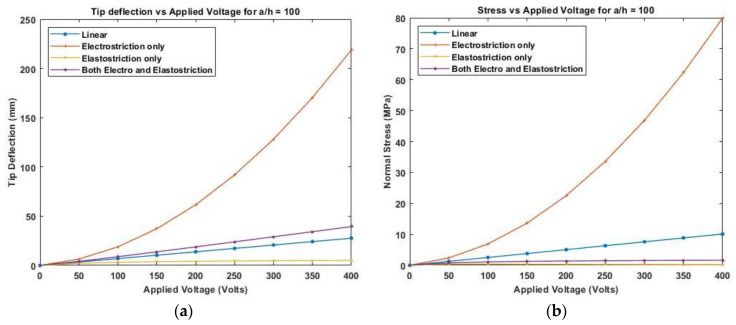
Variation of (**a**) deflection and (**b**) normal stress with electric field for thin anti-symmetric angle-ply laminate.

Similar results are plotted, as presented in Figure 16 and Figure 17, for moderately thick and thin anti-symmetric angle-ply laminated beams. This shows that the consideration of elastostriction in stress calculation is to be considered as the elastic property of the piezoelectric actuator will be affected at a high electric field. 

### 3.4. Analysis of Piezo-Actuated Laminated Composite Beam with Different End Conditions

A piezoelectric symmetric cross-ply and anti-symmetric angle-ply composite laminated thin beam with a thickness ratio of a/h=100 are used to investigate the effect of nonlinear piezoelectric parameters. 

**Figure 18:**
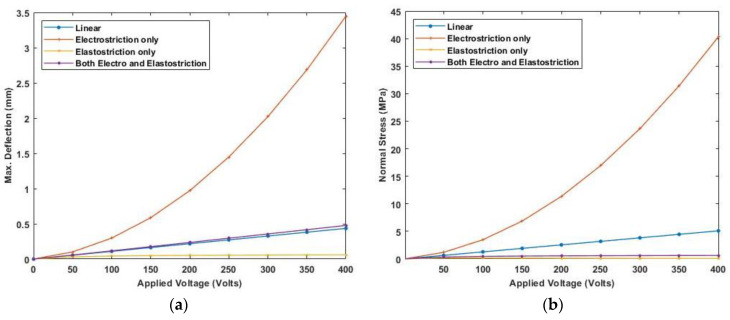
Variation of (**a**) maximum deflection and (**b**) normal stress with electric potential for cross-ply laminate with SS end condition.

**Figure 19:**
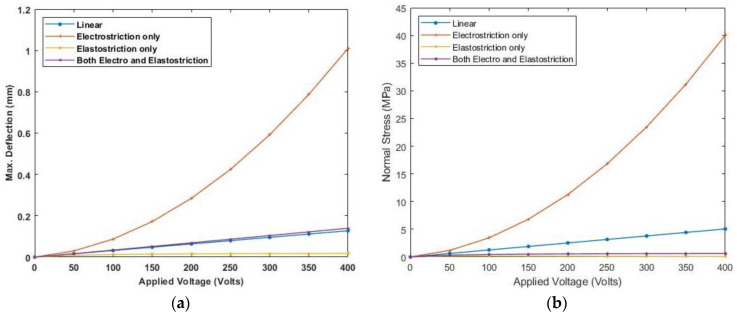
Variation of (**a**) maximum deflection and (**b**) normal stress with electric potential for cross-ply laminate with CS end condition.

**Figure 20:**
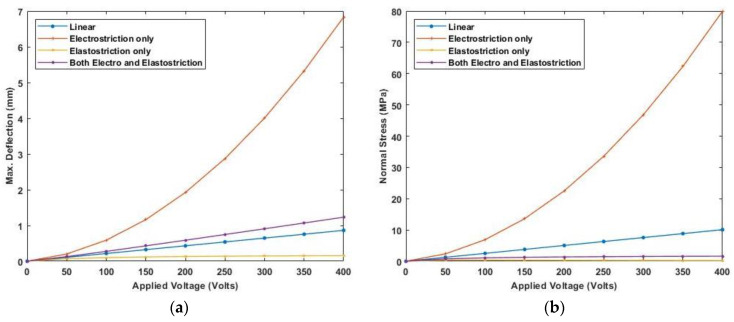
Variation of (**a**) maximum deflection and (**b**) normal stress with the electric potential for anti-symmetric angle-ply laminate for SS end condition.

**Figure 21:**
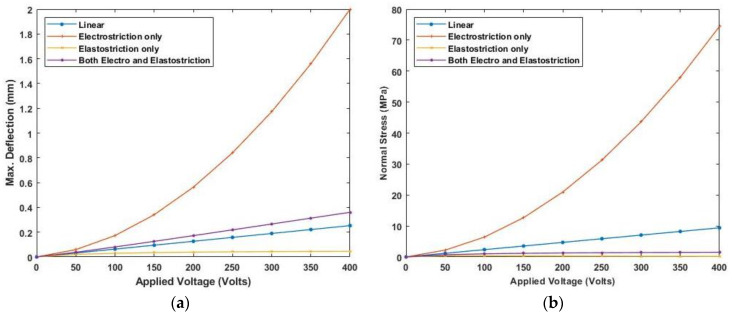
Variation of (**a**) maximum deflection and (**b**) normal stress with the electric potential for anti-symmetric angle-ply laminate for CS end condition.

A varying electric field potential from 0 to 400 V is applied and various end conditions are used to plot the deflection and stress variation. Figure 18 and Figure 19 show maximum deflection and stresses for both ends simply supported (SS) and clamped-simply supported (CS) end conditions using linear and nonlinear piezoelectric constitutive relationships for cross-ply laminate whereas Figure 20 and Figure 21 present the deflection and stress variation for both ends simply supported (SS) and clamped-simply supported (CS) end conditions for anti-symmetric angle-ply.

The results show maximum deflection and stresses are higher for simply-supported beams. Applying a positive electric potential i.e., a negative electric field, smaller values of deflection and stresses are obtained when elastostriction is considered along with electrostriction. Whereas positive electric fields, including the elastostriction, produces more deflection and stresses than electrostriction alone. 

### 3.5. Nonlinear Analysis for Deflection and Stress Distribution of Composite Laminates 

By applying a 400 V to symmetric cross-ply laminate and anti-symmetric angle-ply laminated beam with an aspect ratio of a/h=100, the effect of considering electrostriction coefficient, elastostriction coefficient, and both electrostriction and elastostriction coefficients on transverse deflection, normal stress, and transverse shear stress is illustrated in Table 5 and Table 6 by comparing result using linear piezoelectric constitutive equation. In the case of symmetric cross-ply laminates, the transverse deflection for the linear case is $w\left(0,L\right)=1.7423\;mm$, the normal stress is $\sigma_{xx}=5.0932\;MPa$ at the top of the piezoelectric structure, and shear stress at the mid-plane is $\tau_{zx}=0.248\;MPa.$ Whereas in the case of anti-symmetric angle-ply laminated, the linear deflection is $w\left(0,L\right)=27.6286\;mm$, the normal stress is $\sigma_{xx}=10.0964\;MPa$ at the top of the piezoelectric structure, and shear stress at the mid-plane is $\tau_{zx}=0.212\;MPa.$ Table 5 and Table 6 present the percentage deviation in deflection and stresses when the constitutive equation includes the non-linear coefficients from the linear part. 

**Figure 22:**
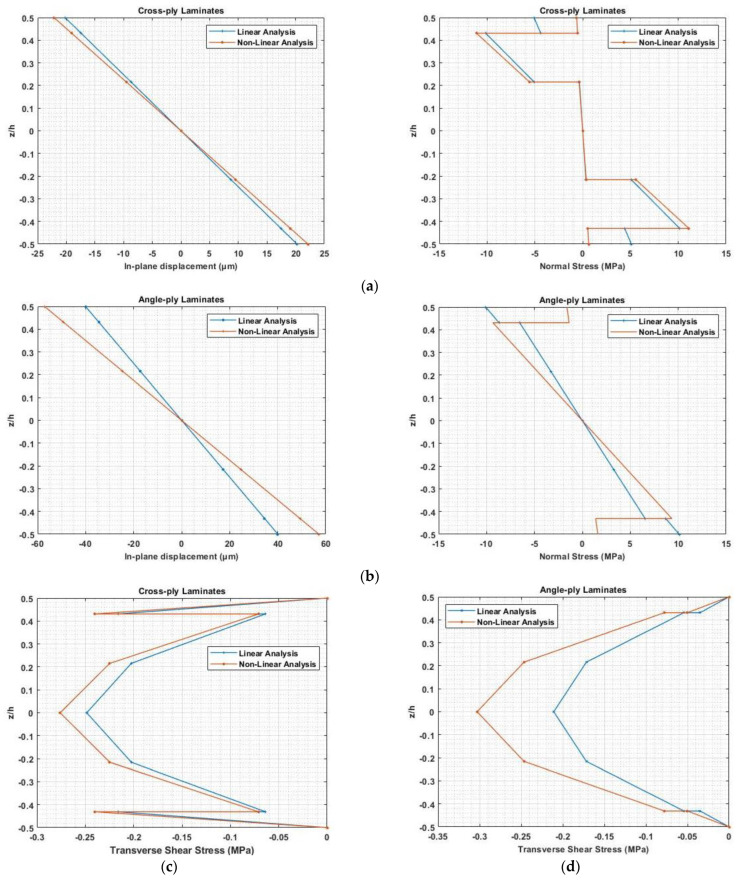
In-plane deflection, normal stress distribution for (**a**) symmetric cross-ply laminate, (**b**) anti-symmetric angle ply laminate and transverse shear stress distribution for (**c**) symmetric cross-ply laminate and (**d**) anti-symmetric angle ply laminate.

Figure 22 presents the in-plane displacement and normal stress distribution along the thickness for symmetric cross-ply and anti-symmetric angle-ply laminated cantilever beams with the applied electric potential of 400 V considering nonlinear elastostriction along with electrostriction coefficient and only linear constitutive equations. The figures illustrate that considering both the elastostriction and electrostriction coefficients results in higher displacement, normal stress, and higher value of transverse shear stress than the linear analysis. In the static analysis of the Piezo-laminated composite beam, the effect of the elastostriction coefficient cannot be ignored. Due to the coupling between extension and bending stiffness, the deflection and stresses are higher in anti-symmetric angle ply laminate than in symmetric cross-ply laminate. 

## 4. Conclusions

This study considers the effect of nonlinear electrostrictive and elastostrictive coefficients in modelling cantilever composite laminated beams using higher-order shear deformation theory. Finite element formulation is used to analyze the effect of nonlinear piezoelectric coefficients on the static analysis of piezoelectric composite laminated beam. A cantilever piezoelectric unimorph is used to validate the nonlinear model. The results show that the nonlinear coefficients for the PZT actuator play a significant role at high electric field and the deflections and stresses cannot be predicted accurately with linear constitutive equations. Under an applied electric field, the electrostriction coefficient increases the deflection and stresses whereas the elastostriction coefficient decreases the deflections and stresses in the piezoelectric structure. The tip deflections and stresses increase with increased applied electric potential when combined nonlinear coefficients are considered. For thick, moderately thick, and thin piezoelectrically actuated laminates considering both nonlinear coefficients, there is a difference in tip deflections from the linear response, and the stresses in piezoelectric actuators are lower than using linear constitutive equations. The nonlinear response is affected by bending stiffness in composite laminated beams, the deviation from the linear response is more when the bending stiffness is less. Due to the coupling between extension and bending stiffness, higher deflection, and stresses are obtained for anti-symmetric angle ply laminates than symmetric cross-ply laminates. The deflection and stress results show nonlinear piezoelectric coefficients cannot be ignored during static analysis of composite laminated beams. The effect of considering both nonlinear terms is needed to be further analyzed for the dynamic analysis of composite laminates.

## Figures and Tables

**Table 1:** Material properties of PVDF actuator.

E (GPa)	G (MPa)	υ	$e_{31}$	$e_{32}$
2.0	775	0.29	0.046 cm^−2^	0.046 cm^−2^

**Table 2:** Transverse deflections of the beam along the length $\left(\times \;10^{-7}m\right)$.

x (m)	FE Tzou/Ye [24]	FE Jiang & Li [4]	FE Present	Theoretical Jiang & Li [4]
0.02	0.132	0.136	0.138	0.138
0.04	0.528	0.545	0.552	0.552
0.06	1.19	1.226	1.242	1.242
0.08	2.11	2.18	2.208	2.208
0.1	3.30	3.41	3.45	3.45

**Table 3:** Material properties ($E_{i},\;G_{ij}\;in\;GPa\;d_{ij}\;in\;10^{-12}\;mV^{-1})$.

Material	$E_{1}$	$E_{2}$	$E_{3}$	$G_{23}$	$G_{13}$	$G_{12}$	$\upsilon_{23}$	$\upsilon_{13}$	$\upsilon_{12}$	$d_{31}$	$d_{32}$
PZT [25]	63	63	63	24.8	24.8	24.8	0.28	0.28	0.28	−166	−166
PZT-4 [26]	81.3	-	64.5	-	25.6	25.6	-	0.43	0.43	−122	−122
Al [26]	68.9	68.9	68.9	27.6	27.6	27.6	0.25	0.25	0.25	-	-
Adhesive [26]	6.9	6.9	6.9	2.46	2.46	2.46	0.4	0.4	0.4	-	-
T300/934 [26]	132.38	-	107.6	-	56.5	56.5	-	0.24	0.43	-	-

**Table 4:** Material properties ($E_{i},\;G_{ij}\;in\;GPa\;d_{ij}\;in\;10^{-12}\;mV^{-1})$.

Material	$E_{1}$	$E_{2}$	$E_{3}$	$G_{23}$	$G_{13}$	$G_{12}$	$\upsilon_{23}$	$\upsilon_{13}$	$\upsilon_{12}$
PZT 3203 HD [11]	60.24	60.24	47.62	19.084	19.084	24.04	0.494	0.494	0.253
AS/3501 Gr/Ep [25]	144.8	9.65	-	5.92	7.1	7.1	-	-	0.3
PZT APC 850 [15]	63	63	63	24.05	24.05	24.05	0.31	0.31	0.31
Silicon [15]	166	166	166	65.9	65.9	65.9	0.26	0.26	0.26
	$d_{31}$	$d_{32}$	$d_{33}$	$d_{331}\;(m^{2}V^{-2})$		$\kappa_{331}\;(m^{3}N^{-1}V^{-1})$			
PZT 3203 HD [11]	−320	−320	650	−520 $\times 10^{-18}$		-			
PZT APC 850 [15]	−175	−175	-	−1210 $\times 10^{-18}$		−6.3 $\times 10^{-17}$			

**Table 5:** Effect of different nonlinear terms in symmetric cross-ply laminates.

	Elastostriction (%)	Electrostriction (%)	Both (%)
Deflection	−86.16	691.41	9.49
Normal Stress	−98.45	691.42	−87.74
Shear Stress	−85.81	691.32	12.23

**Table 6:** Effect of different nonlinear terms in Anti-symmetric angle-ply laminates.

	Elastostriction (%)	Electrostriction (%)	Both (%)
Deflection	−81.97	691.42	42.65
Normal Stress	−97.98	691.42	−84.038
Shear Stress	−82.22	691.36	43.73
